# Machine Learning Methods in Drug Discovery

**DOI:** 10.3390/molecules25225277

**Published:** 2020-11-12

**Authors:** Lauv Patel, Tripti Shukla, Xiuzhen Huang, David W. Ussery, Shanzhi Wang

**Affiliations:** 1 Chemistry Department, University of Arkansas at Little Rock, Little Rock, AR 72204, USA; lhpatel@ualr.edu (L.P.); tshukla@ualr.edu (T.S.); 2 Department of Computer Science, Arkansas State University, Jonesboro, AR 72467, USA; xhuang@astate.edu; 3 Department of Biomedical Informatics, University of Arkansas for Medical Sciences, Little Rock, AR 72205, USA; DWUssery@uams.edu

**Keywords:** machine learning, drug discovery, deep learning, in silico screening

## Abstract

The advancements of information technology and related processing techniques have created a fertile base for progress in many scientific fields and industries. In the fields of drug discovery and development, machine learning techniques have been used for the development of novel drug candidates. The methods for designing drug targets and novel drug discovery now routinely combine machine learning and deep learning algorithms to enhance the efficiency, efficacy, and quality of developed outputs. The generation and incorporation of big data, through technologies such as high-throughput screening and high through-put computational analysis of databases used for both lead and target discovery, has increased the reliability of the machine learning and deep learning incorporated techniques. The use of these virtual screening and encompassing online information has also been highlighted in developing lead synthesis pathways. In this review, machine learning and deep learning algorithms utilized in drug discovery and associated techniques will be discussed. The applications that produce promising results and methods will be reviewed.

## 1. Introduction

Advancements in computational science have accelerated drug discovery and development. Artificial intelligence (AI) is widely used in both industry and academia. Machine learning (ML), an essential component in AI, has been integrated into many fields, such as data generation and analytics. The basis of algorithm-based techniques, such as ML, requires a heavy mathematical and computational theory. ML models have been used in many promising technologies, such as deep learning (DL) assisted self-driving cars, advanced speech recognition, and support vector machine-based smarter search engines [1,2,3,4]. The advent of these computer-assisted computational techniques, first explored in the 1950s, has already been used in drug discovery, bioinformatics, cheminformatics, etc.

**Figure 1:**
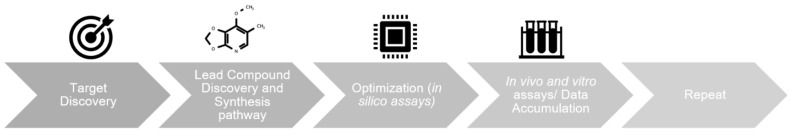
The general steps in drug discovery. Machine learning (ML) and deep learning (DL) algorithms may participate in each of the four steps listed, e.g., by mining proteomic in target discovery, discovering small molecules as candidates in lead discovery, developing quantitative structure-activity relationship models to optimize lead structures for improved bioactivity, and analyzing massive assay results.

Drug discovery has been based on a traditional approach that focuses on holistic treatment. In the last century, the world’s medical communities started to use an allopathic approach to treatment and recovery. This change led to the success of fighting diseases, but high drug costs ensued, becoming a healthcare burden. While quite diverse and specific to candidates, the cost of drug discovery and development has consistently and dramatically increased [5]. As illustrated in Figure 1, the generalized components of early drug discovery include target identification and characterization, lead discovery, and lead optimization. Many computer-based approaches have been used for the discovery and optimization of lead compounds, including molecular docking [6,7], pharmacophore modeling [8], decision forests [9], and comparative molecular field analysis [10]. ML and DL have become attractive approaches to drug discovery. The applications of ML and DL algorithms in drug discovery are not limited to a specific step, but for the whole process. In this article, we review the ML and DL algorithms that have been widely used in drug discovery.

## 2. ML Algorithms Used in Drug Discovery

ML algorithms have significantly advanced drug discovery. Pharmaceutical companies have greatly benefited from the utilization of various ML algorithms in drug discovery. ML algorithms have been used to develop various models for predicting chemical, biological, and physical characteristics of compounds in drug discovery [11,12,13,14,15,16,17,18,19]. ML algorithms can be incorporated in all steps of the process of drug discovery. For example, ML algorithms have been used to find a new use of drugs, predict drug-protein interactions, discover drug efficacy, ensure safety biomarkers, and optimize the bioactivity of molecules [20,21,22,23,24]. ML algorithms that have been widely used in drug discovery, which include: Random Forest (RF), Naive Bayesian (NB), and support vector machine (SVM) as well as other methods [25,26,27].

ML algorithms and techniques are not a monolithic, homogeneous subset of AI. There are two main types of ML algorithms: Supervised and unsupervised learning. Supervised learning learns from training samples with known labels to determine labels of new samples. Unsupervised learning recognizes patterns in a set of samples, usually without labels for the samples. The data are usually transformed into a lower dimension to recognize patterns in high-dimensional data using unsupervised learning algorithms prior to recognizing patterns. Dimension reduction is useful, not only, because unsupervised learning is more efficient in a low dimension space but also because the recognized pattern can be more easily interpreted. Supervised and unsupervised learning can be combined as semi-supervised and reinforcement learning, where both functions can be utilized for various data sets [28]. Expansive volumes of data are critical for the development, evolution, and viability of successful ML algorithms in every step of the drug discovery process. The reliance on big high-quality data and known, well-defined training sets are even more essential in precision medicine and therapies within drug discovery. Precision medicine requires a comprehensive characterization of all related pan-omic data: Genomic, transcriptomic, proteomic, etc., to assist in developing genuinely effective personalized medicines. The widespread use of high-throughput screening and sequencing, online multi-omic databases, and ML algorithms, in the past two decades, have created a flourishing environment for many aspects of data generation, collection, and maintenance required for drug development. The advancements of data analytics have successfully attempted to describe and interpret the generated data. This endeavor, supported with ML techniques and integrated databases through multiple software/web-tools (Table 1, Table 2 and Table 3), is now regularly used for all steps in drug discovery. The ability of new data analytics to synergize with classical approaches and prior hypotheses to produce novel hypotheses and models has proven itself to be useful in applications of repositioning, target discovery, small molecule discovery, synthesis, etc. [29,30,31]. The information generated within the medical and multi-omic fields is multidimensional. The data is often noisy and heterogeneous in character and source. Using ML methods, like generalized linear models through NB, the issues of analysis and interpretation of multidimensional data may be unburdened. Other ML techniques and models commonly used in these areas of analysis include regression, clustering, regularization, neural networks (NNs), decision trees, dimensionality reduction, ensemble methods, rule-based methods, and instance-based methods [31,32].

## 3. Random Forest (RF)

**Figure 2:**
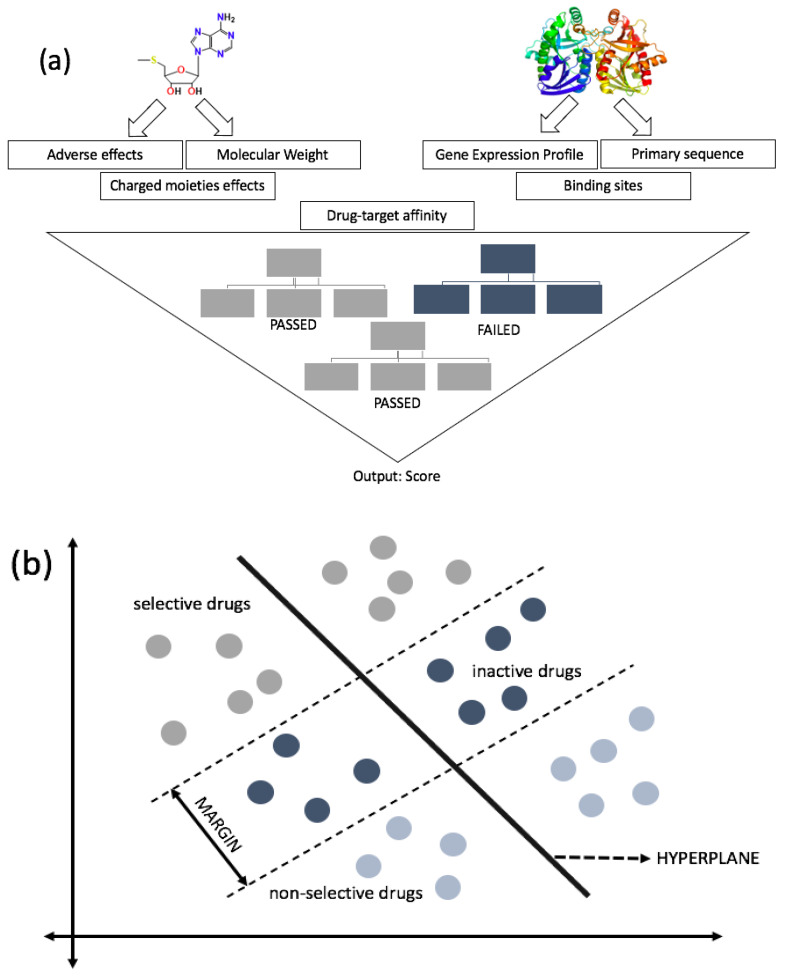
Schematic view of drug development using random forest (RF) (**a**) and support vector machine (SVM) (**b**). (**a**) RF reaches the final decision of drugs by combining the results of randomly-created decision trees (three trees are shown for simplicity). There are multiple features that the computational queries look for in both target and drug. When there is a compatibility match, it proceeds to the next step to match additional features. A series of datasets is inputted into the query, and each tree is responsible for computing a prediction. The prediction picked by most trees is used for the next step. The system of using many decision trees is intended to minimize errors mathematically. (**b**) SVM utilizes similarities between the classes, called support vectors, to distinguish between the classes based on the trained features. It formulates hyperplanes that separate two classes (can be multiclass, if needed). SVM incorporates multiple training sets depending on the classifiers and formulates compounds’ status (active or inactive). During the process, compounds are separated into three sections: Non-selective compounds (active), selective compounds (active), and in the margin are inactive compounds. Although non-selective compounds are active, they are not selective towards the protein of interest. In contrast, selective compounds are active and selective towards the protein of interest.

RF is a widely used algorithm explicitly designed for large datasets with multiple features, as it simplifies by removing outliers, as well as classify and designate datasets based on relative features classified for the particular algorithm. It is commonly trained for large inputs and variables and accessibility based on data collection from multiple databases. It is beneficial in different aspects, such as attributing missing data, working with outliers, and estimating characteristics for classification [25]. The underlying mathematical process of RF consists of several uncorrelated decision trees as an ensemble; each tree is responsible for determining one prediction. The one that constitutes the most votes is considered the best fit (Figure 2a) [36]. Although false positives may happen in any statistical analysis, RF, along with SVM and NB, has been suggested to make the least amount of errors compared to other algorithms. With multiple decision trees, individual errors are minimized due to their assemblies of several predictions rather than solely focusing on one prediction.

In drug discovery, RFs are mainly used either for feature selections, classifiers, or regression. Cano et al. utilized RF methods to improve affinity prediction between ligand and the protein by virtual screening through selecting molecular descriptors, based on a training data set for enzymes, such as ligands of kinases and nuclear hormone receptors. Some of the essential factors accompanying RF in drug discovery are: It expedites the training process, uses fewer parameters, imputes missing data, and incorporates nonparametric data [37]. Rahman et al. utilized multivariate RF by including information relating to genomic sequencing, which helped sustain error and achieve drug responses based on genomic characterizations. Multivariate RFs specialize in limiting error by calculating several error estimates techniques within the system. The computational framework inputs the data that incorporates genetic and epigenetic characterization combinations, allowing the framework to predict the mean and confidence interval of the drug responses. An important quality essential for analyzing any drug to be processed in clinical trials [38]. Rahman et al. endeavored to combine the modeling framework with functional RF for improving the prediction based on the response profile. They tried to combat the difficulties observed in individuals related to finding appropriate compounds depending on individual tumors. RF was incorporated for the generation of the regression tree node and leaf nodes. It acquired the data points of dose-responses. The leaf nodes in the algorithms are responsible for making predictions about the dose-response profile, simultaneously storing the functional data. The model recorded data is comprised of the genome sequences and their characteristics [39]. RF algorithms have also been implemented as a method of classification and regression in a quantitative structure-activity relationship (QSAR) modeling used in lead discovery processes [40,41].

## 4. Naive Bayesian (NB)

NB algorithms are a subset of supervised learning methods that have become an essential tool used in predictive modeling classification. Standard NB algorithms work to classify features of datasets, and depending on the input characteristics, factor correlation, and dimensionality of the data, it can be one of the most efficient techniques for the task [42,43,44]. The effectiveness of NB alongside decision tree algorithms for the use of text mining has not been determined. These techniques enhance the accuracy of retrieved data sets, which generally originate in large, muddled sources [45,46]. Classification of biomedical data is crucial in the drug discovery process, especially in the target discovery subset. NB algorithms have shown great promise as classification tools for biomedical data, often filled with non-related information and data, known as noise [47]. NB techniques could also serve important roles in predicting ligand-target interactions, which could be a massive step forward in lead discovery [48]. Recently, researchers have been able to incorporate NB techniques into diverse applications within the drug discovery process. In a study, Pang et al. used NB models and additional techniques as classifiers for active and inactive compounds, with possible activity as antagonists for estrogen receptors in breast cancer [49]. The researchers utilized the ability of NB algorithms to process vast quantities of information while having a unique tolerance to random noise. The technique, in combination with other tools such as extended-connectivity fingerprint-6, was able to collect excellent outputs. In a recent study, Wei et al. utilized a combinational technique of NB and support vector machine algorithms to predict possible compounds that could be active against targets of human immunodeficiency virus type-1 and the hepatitis C virus generated from multiple QSAR models [50]. Their model utilized NB as a classifier technique paired alongside two different descriptor systems, one also being extended-connectivity fingerprint-6. The utilization of NB, combined with other systems and techniques, has shown to be useful in incorporating drug discovery processes.

## 5. Support Vector Machine (SVM)

SVMs are supervised machine learning algorithms used in drug discovery to separate classes of compounds based on the feature selector by deriving a hyperplane. It utilizes the similarities between classes to formulate infinite numbers of the hyperplane. For linear data, it trains by separating classes consisting of compounds based on selected features and projects them into chemical feature space. An optimal hyperplane attained by maximizing margin between classes in N-dimensional space (N is the number of features); it is denoted by a hyperplane, which is used to classify data points by setting decision boundaries [51]. SVM is crucial to drug discovery because of its capability of distinguishing between active and inactive compounds, ranking compounds from each database (shown in Figure 2b), or training regression model. Regression models are vital in determining the relationship between the drug and ligand, as it employs a query for datasets to predict [52,53,54,55]. When several active compounds are screened against a single protein of interest, SVM can be attributed in various scenarios. SVM classification has a subset binary class prediction that could differentiate between active from inactive molecules.

For drug discovery, it could rank compounds from different databases based on the probability of being active for any computational screening. SVM can be extrapolated in different ways to attain results, with a main focus to distinguish between active and inactive compounds. The process could be manipulated by training the algorithm using various descriptors for feature selectors such as 2D fingerprints, and target protein. A class label is formulated, negative or positive, depending on the direction where the compound is placed from the hyperplane, thereby ranking compounds from the most selective to least selective [55,56]. However, for non-linear data, kernel functions are utilized to optimize the results. Kernel functions plot the data in a higher-dimensional space, where the separation between classes is feasible.

For drug-target interaction, it is specifically designed for integrating ligands and proteins of interest information as an essential component for SVM modeling [51]. Wang et al. investigated drug-target interactions and integrated information obtained from published research of various source to enhance the prediction. They used kernel function to incorporate information on drug pharmacological and therapeutic effects, drug chemical structures, and protein genomic information to characterize the drug-target interactions. Generally, results from the different sources were all promising, and kernel function for the prediction of pharmacological and therapeutic effects displayed the most potential [57]. SVM are also frequently used in predicting drugs that could have multiple bioactivities. For example, Kawaii et al. used SVM classifiers to construct a query where drugs were set against hundreds of targets to establish different biological pathways targeting their bioactivities [58]. In another study, a similar process was used to determine the bioactivities for antihypertensive drugs. The information about the drug activity was obtained from the Market Driven Demand Response database, and a multi-label SVM was employed to produce the query that shows the bioanalysis of drugs [59,60]. Drugs were discovered to be dual inhibitors against both angiotensin-converting enzyme I and neutral endopeptidases.

## 6. Limitations

ML algorithms have been an essential component of drug discovery. These methods increase efficiency and explore thousands of combinations that would have been impossible without this technology. As stated earlier, algorithms are trained with inputted data, but there are a few constraints with this technique. Although ML has been around for quite some time now, the biological pathways/targets being discovered are still novel. Information for the particular protein of interest might be limited, resulting in not much-extrapolated data. Free Energy Perturbation method is a platform where biological information regarding the protein is generated based on computational screening [61]. Data gathered from this method is utilized for training algorithms; however, not all the information is collected from a wet lab, rather computer-generated prediction is utilized. The accuracy of the training data might be lower than anticipated. Even though algorithms discussed in this review have a higher threshold for minimizing errors, there are still some categorical errors from training sets [61].

A more concise way to understand this is by the statistical angle. With algorithms prediction, there is always a concern with overfitting or underfitting. Overfitting is when the model consists of lower quality information/technique but generates higher quality performance. It occurs when the model picks up unusual features during the training, resulting in a negative impact on the model [17]. In contrast, underfitting models fail to recognize the data sets’ underlying trend and generalize the new data inputted [62]. Both underfitting and overfitting result in inaccurate results. There are several ways to tackle overfitting and underfitting, such as increasing the sample size and cross-validation. Cross validation is an often-used technique used to estimate the accuracy of the ML algorithms’ models, by using independent data sets to infer the models.

Another concern raised by chem-informaticians is ample chemical space constructed through algorithms [52,63]. The chemical area is a relative set of descriptors, consisting of thousands of compounds within a frame with boundaries generated by ML algorithms [64,65]. The challenge with chemical space is the clustering of compounds with high density, which often leads to avoidance of compounds with some essential properties and compounds. Studies regarding these issues are discussed later, models to augment chemical space coverage to highlight the molecules with properties different from others [19,66].

## 7. Deep Learning (DL) Methods

DL algorithms are considered one of the cutting-edge areas of development and study in almost all scientific and technological fields. The renaissance of artificial NNs into workable algorithms from their former theorized and predicted applications, first developed in the 1950s, is an essential pillar of DL and the continued success brought by AI-based integration of standard techniques. DL algorithms give computational models the ability to learn a representation of multidimensional data through abstraction [67]. DL has allowed for resolving many challenges faced by standard ML algorithms, including image recognition and speech recognition. In the drug discovery process, DL techniques have become exemplar methods of drug activity prediction, target discovery, and lead molecule discovery [68,69,70]. The basis of DL is often implicated in NN systems, where they are used to create systems that have the capability to complete complex data recognition, interpretation, and generation. The main subsets of artificial NNs used in current drug discovery are deep neural networks (DNNs), recurrent neural networks (RNNs), and convolutional neural networks (CNNs).

**Figure 3:**
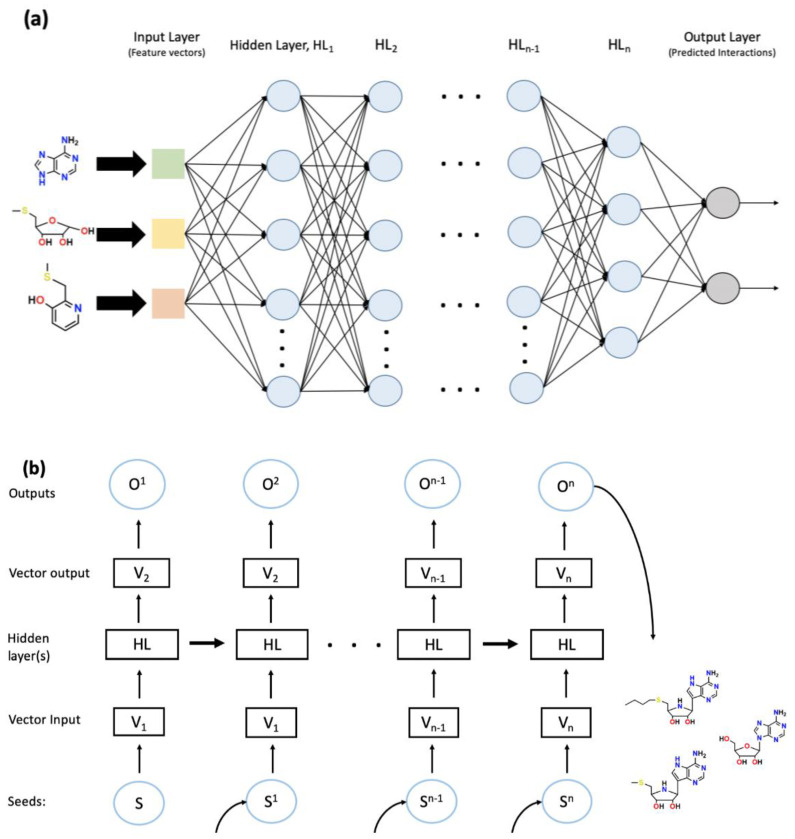
The general scheme of deep neural network (DNN) (**a**) and recurrent neural network (RNN) (**b**). (**a**) DNN consists of an input layer followed by several hidden layers and an output layer. In this case, the input layer utilizes feature vectors generated by a convolutional network. The progression of the NN follows a single path through hidden layer 1 (HL_1_) to HL_n,_ indicating the feedforward nature of the NN. The generated outputs are often processed using supervised learning techniques for the identification and collection of sensible interactions. (**b**) RNN begins with a seed, S, which is inputted into the system. Through the use of algorithmic processing, the seed is turned into a reference vector, V1, which is used by the HL to generate a vector output, V2. V2 is subsequently optimized through input training sets and creates the output, O. The generation of these outputs eventually leads to the creation of a gatherable data set. In the meantime, the HLs feed forward to provide information from previous steps. One example is chemical structure generation using SMILE string characters as seeds; hence the desired gathered outputs would be a string of SMILE characters that would be the desired structure. The dataset created in the figure is gathered and analyzed into the resultant molecules.

The utilization of specific NNs from the variations that exist in the subset is dependent on multiple factors. DNNs, a specific type of feed forward neural networks, function with singular path data flow from the input layer through the hidden layer(s) reaching an output layer (Figure 3a). The outputs generated are typically identified using trained supervised learning algorithms. DL algorithms function through neural networks which can incorporate other ML techniques for training. Through supervised and reinforcement learning guided methods, a DNN can be trained to complete complex tasks. A generative DNN can create novel chemical compounds from existing libraries and training sets (Figure 3a); while, a predictive DNN can predict the chemical attributes of the novel compounds [71,72]. QSAR models are currently being used to find the correlation between these compounds’ chemical structure and activity. QSAR analysis is one of the most advanced forms of DL-based AI in current drug discovery and development. It has allowed researchers to take 2D chemical structures and determine physicochemical descriptors related to the molecule’s activity. 3D-QSAR has allowed further inquiry of geometric structure impacting ligand-target interactions [33,73,74]. QSAR has been actively used in the pharmaceutical industry to predict on/off-target activities of developed lead compounds on specific targets. These algorithmic approaches to discovery and development are not, by all means, full proof or thoroughly capable.

There are always some error sources and imprecision over the multiplicity of studies conducted using these AI algorithms. It has been found that NNs face a few deficiencies in comparison to other ML algorithms in their applications of QSAR studies. The first being the presence of excess descriptors that cause redundancy in NN and eventual clogging of outputs. This redundancy can significantly drop the efficiency of the NN, while also creating non-ideal outputs. Unknown descriptors also pose an issue because they may also affect the output generated. These issues have been alleviated using more specific feature selection algorithms to get a smaller number of higher quality descriptors; however, it will continue to be a problem faced by NN-based QSAR. The second issue with these NN-based assays is implementing ideal network parameters without overfitting [74]. Remedies to this issue have been proposed and implemented, but it persists to be a recurring issue without the necessary adjustments [75].

Once the initial work of target discovery is complete and better understanding is developed for target-molecule interaction, chemical synthesis and characterization become a priority in the pipeline. An important note in this process is using descriptive simplified molecular-input line-entry system (SMILES) nomenclature in much of the algorithms regarding de novo drug design and discovery. RNNs, which are a type of NN that utilize a system of self-learning through generational processing of the inputs and developing hidden layers. The subset RNN-type long short-term memory have become a reliable, standardized method for generating novel chemical structures. RNNs are unique in their ability to use neurons connected in the same hidden layer to form a functioning cycle of processing inputs and outputs compared to DNNs and feedforward neural networks (Figure 3b), which have no connections within the same layer and only push outputs. These generative RNNs have shown promising results in the generation of sensible, structurally correct, and feasible, novel SMILE structures that were not included in the original SMILE training sets [76,77,78,79]. A recent study by Segler et al. used generative RNN models to develop possible molecular structures that could have activity against *Staphylococcus aureus* (*S. aureus*) and *Plasmodium falciparum* (*P. falciparum*). Their models were given small sets of molecular structures that had known activity against these target organisms; from these inputs, the model generated 14% of the 6051 potential molecule candidates for *S. aureus* that has been developed by medicinal chemists. The model also generated 28% of the existing compounds developed for *P. falciparum* [80]. Traditionally, the generation and implementation of chemical synthesis routes have been the sole responsibility of chemists. However, this role is evolving to include more and more computational based synthesis, also known as computer-aided synthesis planning (CASP), with the emergence of AI [81,82,83]. The Monte Carlo tree search (MCTS) based through NN techniques have been used in current studies to generate CASP workflows. The MCTS technique is ideal for this purpose because of the simulation’s ability to perform random continuous step searches without branching until optimal conditions and solutions are met [82,83]. In a groundbreaking study conducted by Segler and Waller [84], an MCTS method using three NNs alongside 12.4 million transformation rules, retrieved through AI-based data mining, from all the available chemical synthesis literature at the time to generate a sensible workflow for CASP. The first NN, an expansion node, retrospectively searches for new transformations to create the molecule; it also predicts the feasibility of applying the transformation from the 12.4 million transformation rules. This allows the expansion node to select the best, as in most feasible and high yielding, transformations from the literature. The second NN, a rollout node, filters the inputs to include only the most frequently reported transformation rules to enable the best possibilities of successful transformations. The update node then incorporates the new pathway into the search tree. This algorithm was able to solve 80% of retrosynthesis problems in just 5 s, and >90% of problems in 60 s [82,83,84]. Various studies have been conducted to optimize AI-based chemical synthesis and reaction routes [85,86,87]. Through the further implementation of AI-based chemical synthesis and characterization, it will be possible to move drug discovery further from the bench to in silico and increase the time and cost-efficiency of discovery and development.

CNNs are a subset of DNNs that take inputs, assign weights to specific parts of the input, then build the ability to differentiate the data. While traditional DNNs are limited in their ability to function correctly on higher-dimensional datasets, CNNs serve as a gleaming solution to tackling this issue with their ability to preserve input dimensionality. The training required for a CNN model is significantly less than DNNs, and RNNs would need to function with reasonable accuracy and efficacy. These advantages have allowed it to become a prominent learning algorithm for image recognition, surpassing other standard ML algorithms. In the process of drug discovery, CNNs have become efficient tools used in target discovery, lead discovery and characterization, in silico target-lead interaction screening, and protein-ligand scoring [68,88,89,90]. Combinations of these DL techniques, such as CNNs, have also been very successful in identifying gene mutations and disease targets [91,92]. The incorporation of CNNs into drug development is not merely limited to target discovery; it has also been widely used in later-stage development. One such use of CNNs in this manner to assist in the generation of motility models of cancer cells responding to treatment [93]. In a recent study, Feng, Zhang, and Shi demonstrated the use of deep learning based drug-drug interaction (DDI) predictors [94], with the aim to address a wet lab issue during the drug discovery, which is often costly and time consuming. The researchers developed a new method utilizing graph convolutional networks and DNN models. In their design, the graph convolutional network served as a structure feature extractor from drugs found in DDI, learning low-dimensional representations (vectors) of the features from the DDI networks. The information is then taken to the DDN model which served as the actual predictor; the ability of the model to take the feature vectors and link them with corresponding feature vectors of possible drug combinations allowed it to produce the interaction prediction. Encouragingly, the predictions using their method outclassed popularly used state-of-the-art-methods [94].

## 8. Examples of Drug Discovery (Paper Summaries and Relevance to Topic)

ML is already being used to develop novel molecules that could be used as future antibiotic candidates. In a recent, groundbreaking study conducted by Stokes et al., the researchers demonstrated the utility and capability of ML techniques in the drug discovery process [95]. They specifically capitalized on the use of DNNs to create novel molecules with broad-spectrum antibacterial activity. These discovered candidates were also identified to be structurally distinct from any known antibiotics. The researchers utilized a training set of 2335 molecules for a DNN model to predict the growth inhibition of *Escherichia coli*, followed by the running of the model on greater than 107 million molecules from several chemical libraries. This gave the researchers the ability to identify potential lead compound candidates that may have similar bioactivity. Through scoring generated by the model, the researchers were able to identify a list of sensible candidates that meet a predetermined score threshold and various other eliminative criteria. The researchers’ efforts proved fruitful, and they were able to identify a c-Jun N-terminal kinase inhibitor, halicin, that is distinct from known antibiotics. This antibacterial candidate was also discovered to be a potent growth inhibitor of *Escherichia coli*, and had shown efficacy against *Clostridioides difficile* and *Acinetobacter baumannii* infections in murine models [95]. In a study conducted by Fields et al., ML algorithms, including NNs-based techniques and SVM models, were used to discover novel antimicrobial peptides, also known as bacteriocins, from bacteria could ultimately be used as compelling antibiotic candidates [96]. Discoveries such as that of the bacteriocins are the outcomes of the machine-learning algorithm’s ability to build and function as complex processing systems. In the study, a positive and negative training set of 346 bacteriocins was used to train the algorithm. These input bacteriocins were represented as complicated vector sums. The machine-learning algorithm then took the inputs and generated new vector structure outputs that preserved the original inputs’ key features. These outputs were translated into 676 bacteriocins that were not identical to the input bacteriocins. From the output bacteriocins, 28,895 peptides were generated using a sliding window algorithm; these peptides spanned 20-mers and were placed through biophysical parameters. Fields et al. then selected 16 of the highest affinity peptides from the biophysical filtration for in vitro testing. Their finding indicated that the peptides had significant antimicrobial activity against *Escherichia coli* and *Pseudomonas aeruginosa* [96].

The utility of ML-based mining has proved to be extremely advantageous with the advent of high throughput data generation and collection. These algorithms have been extensively used alongside the vast data generated utilizing high-throughput sequencing to enhance the target discovery process [15,97]. The innovation of algorithm-assisted data collection and manipulation has already been implemented in emerging research; recently, it has been used to find novel molecular therapeutic targets for aggressive melanoma. Researchers were able to use unsupervised learning techniques through GeneCluster to identify groups of cell lines, one was a primary melanoma group, and the other was an aggressive melanoma group. Through further analysis using supervised learning techniques, the researchers were able to identify invasion-specific genes related to aggressive melanoma [98]. One of the many challenges with cancer treatments is detecting response profiles designed primarily for individual patients. Sakellaropoulos et al. built a network-based framework. They trained a database containing 1001 cancer cell lines, from the Genomics of Drug Sensitivity in Cancer, using DNN to predict drug responses based on gene expressions. The results were evaluated in several clinical cohorts. DNNs are observed to outperform several others in silico screening due to their capability to embrace biological interactions and create models that can capture the biological complexities and accurately predict clinical response with the help of cancer cell baselines. Their model incorporated RF and elastic net (Enet) algorithms to evaluate the DNN model’s results. This framework was only tested on five patients; thus, not much coverage was obtained through this model; therefore, they expanded their study to a more massive sample size. They utilized response data for two drugs: Cisplatin and paclitaxel, and analyzed it with gene expression profiles and patients’ responses to those two drugs gathered from different clinical trials. The study was done on a small scale, implementing DL network training sets and ML algorithms, with a limited amount of knowledge. It is believed that ML could essentially be a powerful tool to assist within the medicinal field, as more data and information are retrieved on patient response profiles [99].

The diseases discussed have been around for a long time, but the emergent need for a treatment for Coronavirus disease 2019 (COVID-19) has stirred up the research world. The pandemic outbreak has caused detrimental effects around the world, but the COVID-19 virus (SARS-CoV-2) is a novel strain of the same species of virus causing the 2003 Severe acute respiratory syndrome (SARS-CoV-1); thus, several studies are incorporating earlier information into supervised ML to quickly find a remedy for this virus [100]. Researchers worldwide are exhausting all available resources, and ML has helped narrow down the drug candidates and minimize clinical trial failure. Kowalewski and Ray developed ML models to help identify effective drugs against 65 human proteins (target) studied to interact with SARS-CoV-2 proteins. As the virus is known to target the respiratory tract, including nasal epithelial cells and upper airway and lungs, they deduce it from inhaling therapeutics to directly target the damaged cells. They assembled 14 million chemicals from ZINC databases and utilized ML models to predict vapor pressure and mammalian toxicity to rank the chemicals and find drugs that share the same chemical space. Their main goal was to establish a short term and long-term pipeline for future purposes. They utilized SVM and RF to create models that could predict drugs and their efficacy. Although most of the researchers focus on a single protein responsible for replication and host entry, it might only allow short term repair. In the long term, Kowalewski and Ray proposed to look into multiple drugs that could potentially target various proteins with diverse biological pathways [101].

## 9. Conclusions

ML-based techniques seek to revitalize the development of drugs. These methods are based on separate applications in target discovery, lead compound discovery, synthesis, protein-ligand interactions, etc. ML applications are paving the way for algorithm-enhanced data query, analysis, and generation. One such example is ML incorporated into target discovery, based heavily on the refinement and search of existing omics and medical data. Through AI integration using ML techniques, viable targets can be found using data clustering, regression, and classification from vast omics databases and sources. Lead compound discovery, e.g., using QSAR, is currently frequently used to develop sensible molecular candidates based on training inputs. Lead compound synthesis has also been expedited with NN-based retrosynthesis algorithms alongside best-chance trees with the input of vast amounts of accumulated data and rules to develop algorithms that can generate synthesis pathways with greater than 90% accuracy in 60 s. Applications of ML in the processes of drug development have been used for some time now. These applications have proven to be steps above previous methods; the development of ML and DL techniques are not all brand new. They have been carefully crafted and developed through decades of research. This curation of function and utility to ML algorithms and techniques has allowed for the continued success and development in drug discovery. Owing to more precise algorithms, more powerful supercomputers, and substantial private and public investment into the field, these applications are becoming more intelligent, cost-effective, and time-efficient while boosting efficacy.

## Figures and Tables

**Table 1:** Databases used for target discovery.

Databases	Specific Information	Ref.
BRENDA http://www.brenda-enzymes.org	Enzyme and enzyme-ligand information source.	[33]
KEGG http://www.genome.jp/kegg	Database containing genomic information for functional interpretation and practical application.	[33]
PubChem https://pubchem.ncbi.nlm.nih.gov	Database for encompassing information on chemicals and biological activities.	[33]
TTD http://bidd.nus.edu.sg/group/ttd/ttd.asp	Therapeutic Target Database containing encompassing information about the drug resistance mutations, gene expressions, and target combinations data.	[33]
DrugBank http://www.drugbank.ca	Detailed drug data and drug-target information database.	[33]
SuperTarget http://bioinfapache.charite.de/supertarget	Drug-related information databases with more than >300,000 compound-target protein relations.	[33]
TDR targets http://tdrtargets.org	Database containing chemogenomic information for neglected tropical diseases.	[33]
STITCH http://stitch-beta.embl.de	Chemical-Protein interaction networks.	[28]
SMD http://genome-www5.stanford.edu	Database of raw microarray datasets.	[34]
Gene Expression Omnibus http://www.ncbi.nlm.nih.gov/geo	Database of raw microarray datasets.	[34]
caArray http://array.nci.nih.gov/caarray	Database of cancer-related microarray datasets.	[34]
CGAP database http://cgap.nci.nih.gov	Database of cancer-related microarray datasets.	[34]
Oncomine http://www.oncomine.org	Database of cancer-related microarray datasets.	[34]
UniHI http://www.unihi.org	Database of human molecular interaction networks.	[34]
Pathguide http://www.pathguide.org	Database of 702 biological pathway related resources and molecular interactions.	[34]
UniProt http://www.uniprot.org	Encompassing protein information center.	[34]
InterPro http://www.ebi.ac.uk/interpro	Database of protein domain information.	[34]

**Table 2:** Web-tools and software utilized in target discovery.

Web-Tools/Software Used for Target Discovery	Specific Information	Ref.
GoPubMed http://www.gopubmed.org	PubMed search engine utilized as a text-mining tool.	[34]
Textpresso http://www.textpresso.org	Full-text engine used in text mining, classification, and search.	[34]
BioRAT http://bioinfadmin.cs.ucl.ac.uk/biorat/docs/index	Full-text search engine used for text mining.	[34]
ABNER http://pages.cs.wisc.edu/~bsettles/abner	Molecular biology text analyzer and entity tagger tool.	[34]
PPICurator https://ppicurator.hupo.org.cn	Tool used for mining comprehensive protein-protein interaction.	[34]
GeneWays http://geneways.genomeleft.columbia.edu	Biological pathway extracting tool.	[34]

**Table 3:** Databases used for lead discovery, optimization, and synthesis.

Database	Specific Information	Ref.
ADReCS http://bioinf.xmu.edu.cn/ADReCS	Database of toxicology information with 137,619 Drug-ADR pairs.	[35]
ChEMBL https://www.ebi.ac.uk/chembl	Database of drug-like small molecules with predicated bioactive properties.	[35]
ChemSpider http://www.chemspider.com	Encompassing database of over 64 million chemical structures.	[35]
DrugCentral http://drugcentral.org	Database containing relevant drug information of activity, chemical identity, mode of action, etc.	[35]
